# ESCRT-III subunits Snf7-1 and Snf7-2 differentially regulate transmembrane cargos in hESC-derived human neurons

**DOI:** 10.1186/1756-6606-4-37

**Published:** 2011-10-05

**Authors:** Jin-A Lee, Lei Liu, Robyn Javier, Anatol C Kreitzer, Celine Delaloy, Fen-Biao Gao

**Affiliations:** 1 Department of Biotechnology, College of Life Science and Nanotechnology, Hannam University, Dajeon 305-811, Korea; 2 Gladstone Institute of Neurological Disease, San Francisco, California, 94158 USA; 3 Department of Neurology, University of Massachusetts Medical School, Worcester, Massachusetts, 01605 USA

**Keywords:** human embryonic stem cells, neurons, ESCRT, CHMP2B, Snf7

## Abstract

**Backgrounds:**

Endosomal sorting complex required for transport (ESCRT) is involved in several fundamental cellular processes and human diseases. Many mammalian ESCRT proteins have multiple isoforms but their precise functions remain largely unknown, especially in human neurons.

**Results:**

In this study, we differentiated human embryonic stem cells (hESCs) into postmitotic neurons and characterized the functional properties of these neurons. Moreover, we found that among the three human paralogs of the yeast ESCRT-III subunit Snf7, hSnf7-1 and hSnf7-2 are most abundantly expressed in human neurons. Both hSnf7-1 and hSnf7-2 are required for the survival of human neurons, indicating a non-redundant essential function. Indeed, hSnf7-1 and hSnf7-2 are preferentially associated with CHMP2A and CHMP2B, respectively, and regulate the turnover of distinct transmembrane cargos such as neurotransmitter receptors in human neurons.

**Conclusion:**

These findings indicate that different mammalian paralogs of the yeast ESCRT-III subunit Snf7 have non-redundant functions in human neurons, suggesting that ESCRT-III with distinct subunit compositions may preferentially regulate different cargo proteins.

## Background

Endosomal sorting complex required for transport ESCRTs (ESCRT-0, ESCRT-I, ESCRT-II, and ESCRT-III) are evolutionarily conserved multiunit proteins that are not only required for many fundamental cellular processes, such as multivesicular body (MVB) formation, cytokinesis, virus budding, and autophagy [1-3]. In yeast, ESCRT-III is composed of four subunits, Vps20, Snf7, Vps2, and Vps24, and the Vps20-Snf7 subcomplex is recruited from the cytoplasm to the endosomal membrane and then bound by the Vps2-Vps24 subcomplex [4]. In contrast, there are three human paralogs of Snf7 (hSnf7-1/CHMP4A, hSnf7-2/CHMP4B, and hSnf7-3/CHMP4C) and two paralogs of Vps2 (CHMP2A and CHMP2B) [3]. However, the functional difference between various ESCRT-III paralogs remains unknown.

ESCRTs are also implicated in several human diseases including neurodegeneration, cancer, and HIV infection [5]. For instance, a splice site mutation in *CHMP2B *is associated with frontotemporal dementia linked to chromosome 3 (FTD3) and produces a mutant protein called CHMP2B^Intron5^, which lacks the C-terminal 36 amino acids [6]. Expression of CHMP2B^Intron5 ^in rodent primary neurons or other cell types blocks the proper dissociation of ESCRT-III, resulting in autophagosome accumulation, dendritic retraction, and eventual neuronal cell loss [7-10]. This neurotoxicity is likely due to enhanced association between the disease protein CHMP2B^Intron5 ^and Snf7-2 [7]. Among ESCRT-III components, Snf7 is thought to play a key role and form oligomers on endosomal membranes [11-13]. Indeed, acute knockdown of Snf7-2 but not CHMP2B in rodent cortical neurons leads to a rapid neuronal cell loss and Snf7-2 knockout mice are embryonic lethal [7].

Oddly, Snf7-1 has not been identified in rodents and its precise functions in neurons remain to be investigated. Here we characterized human postmitotic neurons differentiated from human embryonic stem cells (hESCs) and explored the non-redundant functions of hSnf7-1 and hSnf7-2 in human neurons.

## Methods

### Neuronal differentiation of hESCs

hESCs were differentiated into neurons as described [14]. Briefly, hESC colonies were detached from the feeder layer with 0.5 mg/ml dispase and cultured as aggregates in suspension for 4 days in hESC medium (Dulbecco's modified Eagle medium [DMEM]/12 with 20% knockout serum replacement) without FGF2. Cells were transferred to new culture dishes in the first 2 days to remove adherent MEFs. On day 5, EBs were transferred to flasks precoated with poly-L-ornithine/laminin (20 μg/ml) in neural induction medium (DMEM/F12/N2) consisting of 33% F12, 66% DMEM, 1X N2, 1% NEAA, 10 ng/ml FGF2, and 2 mg/ml heparin. Twelve days after neural induction, neuroepithelial cells in rosettes were isolated from the surrounding cells with 0.2 mg/ml dispase and transferred to new flasks for 2-3 h to allow non-neuronal cells to attach. Floating cells were transferred to a flask coated with poly-HEME (Sigma) to prevent cell attachment.

Terminal differentiation was induced as described [15] with some modifications. At 3-4 weeks of age, neurospheres were dissociated with Accutase (Sigma), plated on glass coverslips coated with poly-D-lysine and laminin (BD Biosciences), and cultured in neurobasal medium supplemented with 2% B27, 1% nonessential amino acids solution, 0.5 mM L-glutamine, 1 μg/ml laminin (Sigma), 10 ng/ml BDNF, and 10 ng/ml GDNF (R&D Systems) for 1-3 weeks. Half of the culture medium was changed every other day.

### Immunocytochemistry

On day 7 or 14 after terminal differentiation, human neurons were fixed with 4% paraformadehyde for 10 min at room temperature (RT) and permeabilized with 0.1% Triton × 100 for 5 min at RT. After blocking with 3% BSA (Sigma), cells were incubated with primary antibodies [anti-nestin (1:100), anti-MAP2 (1:100), anti-BF1 (1:100), anti-GFAP (1:100), anti-PSD95 (1:100)] for 1 h at RT and then with secondary antibodies (anti-mouse or anti-rabbit conjugated with Cy3 or Alexa 488) for 45 min at RT. Anti-NR1 antibody recognizes the synthetic peptide RAEPDPKKKATFRA, corresponding to amino acids 849-862 of NMDAR1.

### Gene expression analysis during neuronal differentiation of hESCs

Total RNA was isolated from human neurons at 3 or 14 DIV and incubated with DNase I for 30 min at 37°C. Reverse transcription was performed with Taq-Man reagent (Applied Biosystem). qRT-PCR was performed with an ABI Prism 7700 Thermocycler with fluorescence detection (Applied Biosystems) as described [14]. The primers were Snf7-1 (sense 5'-cctatgggctttggagatga-3', antisense 5'-ttgtcgcccacatttaacaa-3'); Snf7-2 (sense 5'-cgaaacctgtagggtttgga-3', antisense 5'-ctgtttcgggtccactgatt-3'); Snf7-3 (sense 5'-gaactccacagcaatgagca-3', antisense 5'-ccagcatctcctcagtctcc-3'); NR2B (sense 5'-tctttggagatggggagatg-3', antisense 5'-tcgcagatgaaggtgatgag-3'); mGluR1 (sense 5'-ggaagggaattatggggaga-3', antisense 5'-tgtcatgccttcacaggagc-3'). The primers for GAPDH (Gene ID: NM_00204.3) were from ABI.

### Electrophysiology of human neurons

Cells targeted for whole-cell patch-clamp recordings were visualized with infrared differential interference contrast microscopy (Olympus BX51WI). Cells were immersed in a HEPES-buffered saline solution containing 115 mM NaCl, 2 mM KCl, 10 mM HEPES, 3 mM CaCl_2_, 1.5 mM MgCl_2_, and 10 glucose. All experiments were performed at RT. Resistance of micropipettes was 2.5-4.0 MΩ when filled with the following: 130 mM KMeSO_3_, 10 mM NaCl, 2 mM MgCl_2_, 0.16 mM CaCl_2_, 10 mM HEPES, and 0.5 mM EGTA. Current- and voltage-clamp recordings were obtained with a Multiclamp 700B amplifier (Molecular Devices), filtered at 2 kHz, and digitized at 10 kHz. All data were acquired and analyzed with custom programs written in Igor Pro.

### Induction of depolarization of human neurons

To induce depolarization, human neurons (14-21 DIV) were incubated in neurobasal medium (Invitrogen) with 1 μM tetrodotoxin (Sigma) and 10 μM 2,3-dihydroxy-6-nitro-7-sulfamoyl-benzo[f]quinoxaline-2,3-dione (Sigma) for 30 min at 37°C. In some experiments, 2X depolarization solution (10 mM HEPES pH 7.4, 1.8 mM CaCl_2_, 110 mM KCl, 0.925 mM NaH_2_PO_4_, 0.39 mM MgSO_4_, 5.36 mM NaCl) was added. Depolarization-induced gene expression was determined by qRT-PCR.

### Gene knockdown in human neurons

For generation of virus, each RNA was subcloned into the pSicoR vector (McManus lab, University of California, San Francisco). To knock down *hSnf7-1 *and *hSnf7-2 *in human neurons, specific sequences were selected as targets of siRNA (Additional file 1). For immunoblot analysis, proteins extracts were separated by SDS-PAGE and transferred to a PDVF membrane. After blocking with 5% non-fat milk in 1% Tween 20 in PBS, the membrane was incubated first with primary antibody at 4°C overnight and then with HRP-conjugated anti-mouse or anti-rabbit secondary antibody at RT for 1 h.

### Survival assay for human neurons

To investigate the effect of loss of hSnf7-1 or hSnf7-2 in human neurons, we infected mature cortical neurons (14-16 DIV) with lentivirus expressing siRNA. PI- and GFP-positive neurons were counted as surviving neurons. Three independent cell survival assays were done.

## Results

### Differentiation and characterization of human postmitotic neurons derived from hESCs

**Figure 1:**
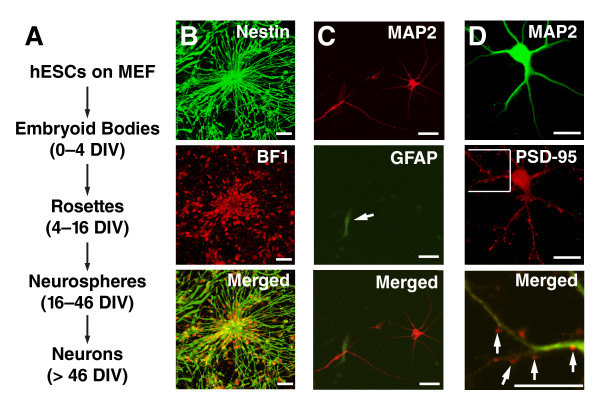
**Differentiation of human postmitotic neurons derived from hESCs**. (A) Differentiation of H9 hESCs into neurons. hESCs (line H9) were dissociated from feeder cells and cultured in aggregates to develop into EBs, which were transferred to flasks containing neural induction medium. Rosette structures were formed and, 12 days after neural induction, were isolated from the surrounding cells to develop into neurospheres. One month later, terminal neuronal differentiation was induced in the presence of BDNF and GDNF. (B) Human neural progenitors at the rosette stage were immunostained with anti-nestin antibody (green) and anti-BF1 antibody (red). Bars: 50 mm. (C) Terminally differentiated human neural cells were immunostained with anti-MAP2 antibody (red) and anti-GFAP antibody (green). Bars: 50 μm. (D) Human neurons derived from hESCs were immunostained with anti-MAP2 antibody (green) and anti-PSD-95 antibody (red). The merged image shows an enlarged image of the boxed area. Bars: 20 μm.

To establish a human cellular model of neurodegeneration, hESCs (H9 cells) were differentiated into postmitotic neurons as previously reported [14]. Briefly, hESCs were grown on mouse embryonic fibroblasts (MEFs) in the presence of fibroblast growth factor 2 (FGF2). Embryoid bodies (EBs) formed in 4 days from dissociated hESCs in the absence of FGF2 and were differentiated in the presence of FGF2 and N2 supplement into a monolayer containing rosette structures. Rosette cells were isolated and placed in suspension cultures to allow human neural progenitor cells (hNPCs) in neurospheres to proliferate for a month. hNPCs dissociated from neurospheres were differentiated into postmitotic neurons and glia in the presence of brain-derived neurotrophic factor (BDNF) and glial cell line-derived neurotrophic factor (GDNF) (Figure 1A).

To use human postmitotic neurons as a cellular model to understand molecular pathogenesis of FTD, we used the protocol described above to differentiate hESCs into postmitotic neurons with characteristics of forebrain neuronal lineages. For instance, hNPCs in rosette structures express nestin and BF1, a transcription factor expressed in telencephalic neural progenitor cells (Figure 1B). hNPCs in neurospheres also express Pax6, a transcription factor containing a paired domain and a paired-type homeodomain involved in early telencephalon patterning [16]. After terminal differentiation, 70-80% of cells were MAP2-positive neurons, with a few GFAP-positive astrocytes (Figure 1C). PSD-95-immunoreactive puncta were formed on dendrites of these human postmitotic neurons (Figure 1D), suggesting the formation of potential synapses.

**Figure 2:**
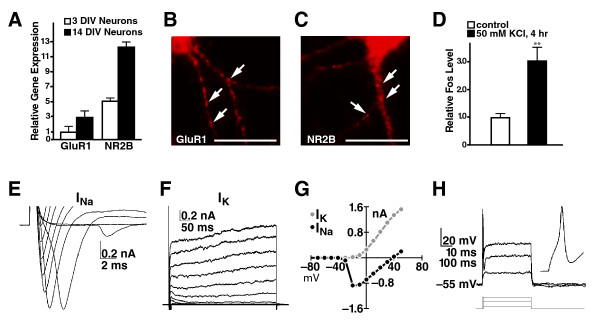
**Characterization of human neurons derived from hESCs**. (A) Expression of NR2B or GluR1 in human postmitotic neurons was confirmed by qRT-PCR analysis. Values are mean ± s.e.m. (B) Immunostaining of neurons at 14 DIV with anti-GluR1 antibody. Bar: 20 μm. (C) Immunostaining of neurons at 14 DIV with anti-NR2B antibody. Bars: 20 μm. (D) Membrane depolarization by 50 mM KCl treatment increased *c-fos *expression in hESC-derived human neurons as measured by RT-PCR analysis. Values are mean ± s.e.m. (E-H) Electrophysiological properties of human neurons derived from hESCs. Voltage-sensitive sodium and potassium currents measured in voltage-clamp (E-G) support the generation of action potentials in response to current injection (H).

These neurons expressed receptors for neurotransmitters, such as glutamate receptor 1 (GluR1) and NMDA receptor 2B (NR2B), as measured by quantitative real-time polymerase chain reaction (qRT-PCR) (Figure 2A) or immunostaining with receptor-specific antibodies (Figure 2B and 2C). In the presence of 50 mM KCl, *c-fos *mRNA expression increased markedly considerably (Figure 2D), indicating that depolarization at the membrane can be coupled with gene expression in the nucleus of these neurons.

These human postmitotic neurons displayed electrophysiological properties similar to those of rodent neurons in primary cultures. In voltage-clamp studies, a series of depolarizing voltage steps elicited an inward Na^+ ^current that rapidly inactivated (Figure 2E) and a longer sustained outward current mediated predominately by K^+ ^(Figure 2F). Current-voltage plots for peak Na^+ ^and K^+ ^currents revealed voltage-sensitive ion channels activating in the range of -40 to -20 mV. To confirm that these membrane conductances support action potential generation, we performed current-clamp recordings (Figure 2G). Neurons exhibited negative resting membrane potentials in the physiological range (-57.5 ± 2.2 mV, n = 4), and action potentials were observed in all four cells tested. For the human neurons, positive current injection elicited an action potential (see inset) followed by sustained depolarization and inactivation of further spiking (Figure 2H). These results indicate that these hESCs-derived human postmitotic neurons have molecular and electrophysiological characteristics similar to those of native neurons.

### hSnf7-1 and hSnf7-2 are expressed in human postmitotic neurons

**Figure 3:**
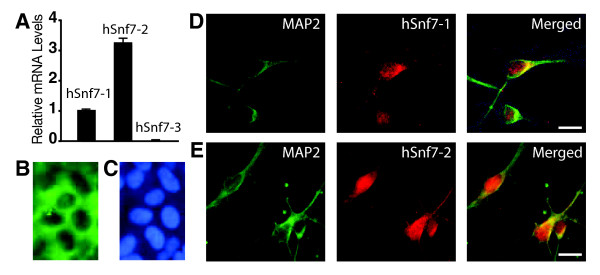
**hSnf7-1 and hSnf7-2 are expressed in human post-mitotic neurons**. (A) qRT-PCR showed that hSnf7-2 was highly expressed in human neurons, while hSnf7-3 was barely detectable. (B) hESCs were immunostained with hSnf7-2. hSnf7-2 was mostly localized to cytoplasm. (C) Nucleus staining (indicated by DAPI staining) of hESCs as shown in panel B. (D) Immunostaining showed that hSnf7-1 was mostly expressed in the cytoplasm of human neurons. Bar: 20 mm. (E) Immunostaining with a hSnf7-2-specific antibody reveals hSnf7-2 in both the nucleus and cytoplasm of human neurons. Bar: 20 μm.

Snf7 is a key component of yeast ESCRT-III [1]. One Snf7 homolog (Shrub) is found in *Drosophila*, and two (mSnf7-2/CHMP4B and mSnf7-3/CHMP4C) are found in mice [17]. Enhanced association was observed between mSnf7-2 and CHMP2B^Intron5^, a mutant protein implicated in FTD3 [7]. In humans, three Snf7 homologs have been reported (hSnf7-1/CHMP4A, hSnf7-2/CHMP4B, and hSnf7-3/CHMP4C)[3]. However, the mouse counterpart of hSnf7-1/CHMP4A has not been identified. Since the rodent homolog for hSnf7-1 has not been identified, the roles of hSnf7-1 and hSnf7-2 in neurodegeneration could only be studied in human neurons. To characterize the functions and contributions of Snf7 family proteins to neurodegeneration in hESCs-derived human neurons, we first compared the relative expression levels of human *Snf7 *family genes by qRT-PCR in human neurons cultured for 7 days in vitro (DIV). *hSnf7-2 *mRNA was three times more abundant than *hSnf7-1*, but the amount of *hSnf7-3 *mRNA was <0.1% of *hSnf7*-1 mRNA (Figure 3A). Thus, hSnf7-1 and hSnf7-2 are major Snf7 family proteins expressed in human postmitotic neurons. Immunostaining revealed hSnf7-2 in the cytoplasm in hESCs (Figure 3B). Both hSnf7-1 and hSnf7-2 were expressed in human postmitotic neurons and seemed to be present in both the nucleus and cytoplasm suggesting that they must have some roles in human neurons (Figure 3D, E).

### Both hSnf7-1 and hSnf7-2 are essential for the survival of human postmitotic neurons

**Figure 4:**
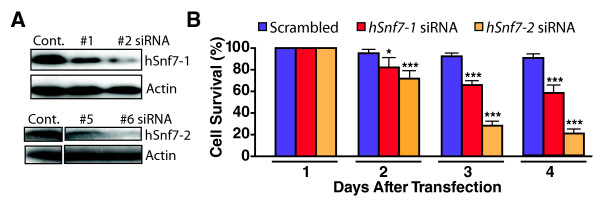
**Both hSnf7-1 and hSnf7-2 are essential for the survival of human neurons**. Terminally differentiated human neurons (14-16 DIV) were infected with lentivirus expressing either *hSnf7*-1 or *hSnf7*-2 siRNA. (A) Western blot analysis showed hSnf7-1 and hSnf7-2 specifically their expression. (B) Infection of lentivirus expressing either *hSnf7*-1 or *hSnf7*-2 siRNA caused human neuronal cell loss, respectively. GFP-positive and PI-negative neurons were considered as surviving neurons.

To dissect the functions of hSnf7-1 and hSnf7-2 in human postmitotic neurons, we generated *hSnf7-1*- or *hSnf7-2*-specific siRNAs and checked efficiency of their knockdown in HEK293 cells (Table S1). We chose one siRNA with most strong knockdown-effect among siRNAs of hSnf7-1 or hSnf7-2 and generated lentivirus constructs expressing *hSnf7-1*- or *hSnf7-2*-specific siRNAs. At 21 DIV, fully differentiated human mature neurons were transfected with *hSnf7-1 *siRNA lentivirus (#2) or *hSnf7-2 *siRNA lentivirus (#6). Each siRNA specifically reduced gene expression of each paralog in human neurons (Figure 4A). Interestingly, reduced expression of hSnf7-1 or hSnf7-2 caused significant neuronal cell loss by days 3 and 4 after infection, suggesting non-redundant functions for this family of ESCRT-III components (Figure 4B). This result implicates that both hSnf7-1 and hSnf7-2 are required for human neuronal cell survival and exert non-redundant functions.

**Figure 5:**
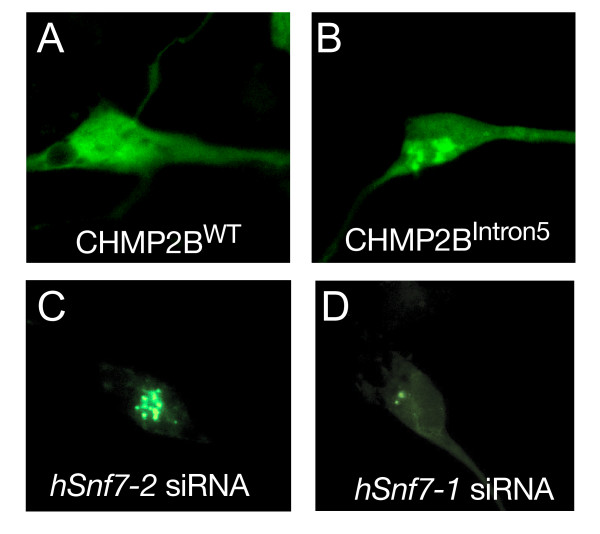
**Autophagosome formation in hESC-derived human neurons**. (A) A CHMP2B^WT^-transfected human neuron. (B) Autophagosomes accumulate in CHMP2B^Intron5^-transfected human neurons, as indicated by GFP-LC3. (C) SiRNA knockdown of hSnf7-2 in human neurons leads to autophagosome accumulation, as indicated by GFP-LC3. (D) SiRNA knockdown of hSnf7-1 in human neurons leads to autophagosome accumulation, as indicated by GFP-LC3.

Consistent with earlier reports on mSnf7-2 [6,7], loss of hSnf7-2 in human neurons also led to accumulation of autophagosomes, as indicated by GFP-LC3 puncta, a phenotype similar to that caused by the expression of the FTD3-associated mutant protein CHMP2B^Intron5 ^(Figure 5B, C). Accordingly, loss of hSnf7-1 by siRNA caused a similar cellular phenotype in human neurons (Figure 5D), again indicating that hSnf7-1 and hSnf7-2 play non-redundant roles in ESCRT-III related cellular processes.

### hSnf7-1- or hSnf7-2-containing ESCRT-III differentially regulates the turnover of distinct transmembrane cargos in human neurons

**Figure 6:**
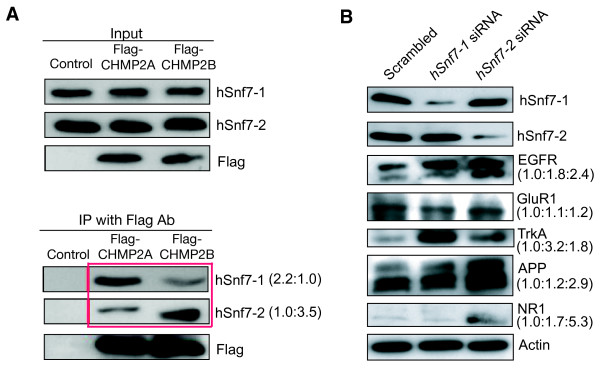
**Differential regulation of diverse transmembrane cargos in human neurons by ESCRT-III with different subunit compositions**. (A) Preferential interactions between hSnf7-1 and CHMP2A and between hSnf7-2 and CHMP2B. Flag-tagged CHMP2A or CHMP2B was transfected into HEK293T cells, and endogenous hSnf7-1 and hSnf7-2 proteins were detected by western blot with specific antibodies after IP with anti-Flag antibody. (B) siRNA knockdown of hSnf7-1 or hSnf7-2 in human neurons had differential effects on the accumulation of different transmembrane cargos. Human neurons were collected 3 days after infection with lentivirus expressing *hSnf7*-1 or *hSnf7*-2 siRNA. Actin was used as the loading control.

To further explore the functional differences between hSnf7-1 and hSnf7-2, we determined whether hSnf7-1 and hSnf7-2 are present in the same ESCRT-III. Among the components of yeast ESCRT-III, only Vps2 has more than one human homolog (CHMP2A and CHMP2B); vps20 and vps24 have only one each (CHMP6 and CHMP3) [3,18,19]. Therefore, we examined the biochemical interactions between human homologs of Snf7 and Vps2 family proteins. Flag-CHMP2A or Flag-CHMP2B was expressed in HEK293T cells. Two days later, immunoprecipitation (IP) was performed with anti-Flag antibody, and immunoisolates were analyzed by western blot with antibodies against hSnf7-1 or hSnf7-2. Intriguingly, endogenous hSnf7-1 preferentially associated with CHMP2A, and complexes containing hSnf7-2 and CHMP2B were more abundant than those containing CHMP2A (Figure 6A). Thus, hSnf7-1 and hSnf7-2 may have preferred interacting partners to form distinct ESCRT-III with different cellular functions.

To test this hypothesis, we examined whether hSnf7-1- or hSnf7-2-contaning ESCRT-III regulates the trafficking of different transmembrane cargos in the MVB pathway. To this end, we examined the degradation and accumulation of different transmembrane cargos as a result of loss of hSnf7-1 or hSnf7-2 activity in mature hESC-derived human postmitotic neurons. The cells were infected at 21-23 DIV with lentivirus expressing *hSnf7-1 *or *hSnf7-2 *siRNA. These siRNAs specifically reduced endogenous hSnf7-1 or hSnf7-2 in human neurons (Figure 4A).

We then examined the protein levels of some receptors essential for neuronal cell survival and signaling in human neurons 2-3 days after lentiviral infections. Reduced expression of hSnf7-1 or hSnf7-2 led to accumulation of epidermal growth factor receptor (EGFR) but did not affect the level of GluR1 (Figure 6B). Interestingly, the nerve growth factor (NGF) receptor TrkA accumulated to a greater extent after knockdown of *hSnf7-1 *than of *hSnf2*. In contrast, amyloid precursor protein (APP), a transmembrane protein implicated in Alzheimer's disease, and NMDA receptor 1 (NR1) accumulated more after knockdown of *hSnf7-2 *(Figure 6B).

## Discussion

Our findings as reported here suggest that different ESCRT-III subunits may associate with each other preferentially to form ESCRT-III with unique subunit compositions and distinct cellular functions in human neurons.

ESCRT machinery is composed of four heteromeric protein complexes, ESCRT-0, -I, -II, and -III [1-3]. Intriguingly, in contrast to yeast ESCRT subunits, many mammalian ESCRT proteins have multiple isoforms such as Snf7-1/Snf7-2/Snf7-3 (CHMP4A/CHMP4B/CHMP4C), CHMP2A/CHMP2B, and VPS37A/VPS37B/VPS37C/VPS37D [1-3,12]. Although they are highly conserved, the significance and unique functions of individual isoforms are largely unknown. As shown in our study, hSnf7-1 and hSnf7-2 but not hSnf7-3 are mostly expressed in human postmitotic neurons and our loss of function studies revealed their functional non-redundancy. The preferential association of hSnf7-1 with CHMP2A and hSnf7-2 with CHMP2B supports the idea that multiple isoforms of ESCRT proteins may form different ESCRT complexes. Indeed, a recent study by Stefani et al. [20] showed that VPS37A but not VPS37C formed endosome-specific ESCRT-I complex with TSG101 and UBAP1. It remains to be determined what are the exact compositions of distinct ESCRT-III complexes and how different transmembrane cargos are differentially recognized in different mammalian cell types and various cellular processes.

In our study, we used the protocol to differentiate hESCs into human postmitotic neurons with characteristics of forebrain neuronal lineages [14-16]. Differentiated human postmitotic neurons have some similar morphological and electrophysiological properties with those of rodent neurons as shown in Figure 2. Thus, this can be a valuable model system to examine human-specific gene functions in neurons and to investigate the molecular pathogenic mechanisms of several neurodegenerative diseases such as FTD.

## Conclusions

In this study, we investigated the molecular and cellular functions of Snf7 family proteins using hESC-derived human neurons. hSnf7-1 and hSnf7-2 are most abundantly expressed in human neurons and both are required for neuronal survival, suggesting non-redundant cellular functions. Indeed, these two paralogs preferentially form complexes with CHMP2A and CHMP2B, respectively, and seem to differentially regulate the turnover of various transmembrane cargos. Thus, unlike in yeast, different ESCRT-III complexes with unique subunit compositions are likely present in human neurons and fulfill specific cellular functions.

## Competing interests

The author declares that they have no competing interests.

## Authors' contributions

JAL and FBG designed the experiments, analyzed the data, and wrote the manuscript. JAL and LL performed most of the experiments. RJ and AK performed the electrophysiological experiments. C.D. was involved in establishing hESC cultures. All authors read and approved the final manuscript.

## Supplementary Material

Additional file 1 **Target sequences in *hSnf7-1 *and *hSn7-2 *for RNAi**. This file contains primer sequences used to generate *hsnf7-1 *and *hsnf7-2*-specific RNAi constructs. Click here for file
