# Supplementary material for: ESCRT-III subunits Snf7-1 and Snf7-2 differentially regulate transmembrane cargos in hESC-derived human neurons
Source: Mol Brain. 2011 Oct 5;4:37. doi: 10.1186/1756-6606-4-37 (PMC3197483; doi:10.1186/1756-6606-4-37)
Supplement: Additional file 1 — Target sequences in hSnf7-1 and hSn7-2 for RNAi. This file contains primer sequences used to generate hsnf7-1 and hsnf7-2-specific RNAi constructs. [file 1756-6606-4-37-S1.PDF]

**Additional file 1**. Target sequences in *hSnf7-1* and *hSn7-2* for RNAi.

***Target sequence for hSnf7-1***

#1: 5’-aagagatgttaagcaaga-3’

#2: 5’-gacatcgataaagttgat-3’

#3: 5’- aattaagttccaactata-3’

#4: 5’-gaacaggaggaactagaca-3’

#5: 5’-ggagatttcaacagcaatt-3’

#6: 5’-ggaattagaagaactagaa-3’

#7: 5’-gaaacgtgcatacattta-3’

#8: 5’-gaaacagtccctctaccaa-3’

***Target sequence for hSnf7-2***

#1: 5’-gaaggggaagaaggagaa-3’

#2: 5’-aaggggaagaaggagaaa-3’

#3: 5’- ggcgacaaggaagaagaa-3’

#4: 5’-aagaagaaccctcagtca-3’

#5: 5’-gatgaagatgaagaagca-3’

#6: 5’-cagaagtccttcgtacca-3’

#7: 5’-ggtagatgaactgatgact-3’

#8: 5’-gattcaacaggagctacaa-3’
